# The analysis of groundwater nitrate pollution and health risk assessment in rural areas of Yantai, China

**DOI:** 10.1186/s12889-020-08583-y

**Published:** 2020-04-03

**Authors:** Guimei Yu, Jiu Wang, Lei Liu, Yun Li, Yi Zhang, Songsong Wang

**Affiliations:** 1 Yantai Center for Disease Control and Prevention, Yantai, Shandong 264003 People’s Republic of China; 2 School of Public Health, Binzhou Medical College, Yantai, Shandong 264003 People’s Republic of China

**Keywords:** Nitrate, Daily intake, Health risk assessment, Hazard quotient

## Abstract

**Background:**

Nitrate is one of the most common chemical contaminants of groundwater, and it is an important unqualified factor of rural groundwater in Yantai. In order to assess the risk of exposure to drinking water nitrate for adults and juveniles, in recent years, we monitored the nitrate concentrations in rural drinking water,a model was also used to assess the human health risk of nitrate pollution in groundwater.

**Methods:**

From the year 2015 to 2018, the drinking water in rural areas of Yantai was tested according to the “Sanitary Standard for Drinking Water” (GB5749–2006). The principal component analysis was used to analyze the relationship between groundwater chemicals and nitrate. The model was used to assess human health risks of groundwater nitrate through the drinking water and skin contact.

**Results:**

A total of 2348 samples were tested during the year 2015–2018.Nitrate and total dissolved solids, total hardness, chloride are all relevant, the above indicators may come from the same source of pollution; The median nitrate content (C_EXP50_) was 17.8 mg / L; the risk of exposure in each group was ranked as: Juveniles > Adult female > Adult male;the median health risk (HQ_50_) for minors and adults exceed 1.

**Conclusions:**

The concentrations of nitrate is stable and does not change over time. The high concentration of nitrate in rural areas of Yantai may be the result of the interaction of fertilizers and geological factors. The risk of exposure to nitrate in juveniles and adults is above the limit, so it is necessary to be on the alert for the high levels of nitrate.

## Background

In nature, inorganic nitrogen exists in the form of nitrates, nitrites and ammonia nitrogen. Among them, nitrite and ammonia nitrogen are unstable, their concentration is low and easily converted to nitrate [1]. Studies in recent years [2] have shown that high nitrate levels in drinking water during the first trimester of pregnancy are associated with birth defects in newborns [3], methemoglobinemia is directly related to high nitrate levels in drinking water [4].

Nitrate may be one of the various factors that cause disease. It is generally believed that nitrate plays a significant role in the occurrence of methemoglobinemia. In order to ensure that the quality of drinking water meets the health needs of human beings, China National Drinking Water Hygiene Standard GB5749–2006 stipulates that the standard limit of nitrate nitrogen in groundwater is 20 mg/L.The World Health Organization’s limit for nitrate nitrogen is 11 mg/L.

Wells are the main source of drinking water in rural areas of many developing countries, these groundwater sources are often untreated. Drinking water of groundwater is an important source of nitrate. There are many sources of groundwater nitrate, such as improper disposal of waste, waste from animal farms [5], use of nitrogenous fertilizers, [6] vegetables (such as Chinese cabbage, kale and carrots), etc. Early studies have shown that nitrate pollution in groundwater may originate from several different sources, including point source pollution (such as industrial pollution and intensive animal husbandry) and non-point source pollution (such as fertilizers, fungicides, atmospheric deposition, etc.) [7]. Groundwater pollution is very complex and often difficult to observe and has long-term effects [8]. Compared with surface water, once groundwater is polluted by some substances, it is difficult to recover [9].

Rahmati et al. (2014) showed that the amount of nitrogen fertilizer applied was positively correlated with nitrate concentration of groundwater [9]. Babiker et al. (2004) used the Geographic Information System (GIS) to study the nitrate pollution in groundwater,it was found that nitrate content in groundwater of vegetable-growing farmland increased significantly due to the large-scale use of chemical fertilizers [10]. Excessive use of chemicals and fertilizers [11] in agriculture can increases the risk of surface and groundwater pollution, which has adverse effects on human health and the environment [12].

In Yantai, most of the residents in rural areas use underground well water as drinking water sources, and most of underground well water don’t have any treatment. Monitoring results of centralized water supply in rural areas of China show that nitrate nitrogen is an important unqualified factor. In China, the research of most HHRA models focuses on heavy metals, organic pollutants and pesticides.

This article is dedicated to studying the causes of high nitrate concentrations in drinking water in rural areas of Yantai, and the possible harm index of exposure to this concentration of nitrate to the residents of Yantai.

## Methods

### Study design

From 2015 to 2018, the Yantai Center for Disease Control and Prevention (CDC) selected rural drinking water (well) monitoring sites, and the county-level centers for disease control and prevention took charge of monitoring tasks, while the Yantai CDC was responsible for quality control. The monitoring frequency is twice a year, once in the dry season (March to April) and the other in the rainy season (July to August). The collection, storage, transportation and inspection of water samples was carried out in accordance with the Standard Test Method for Drinking Water of China (GB/T 5750–2006).

All levels of CDC laboratories participating in the monitoring work are qualified for nitrate detection (all have passed CMA metrological certification). Quality control covers the entire process of sample collection, storage, transportation, and laboratory testing. Prior to laboratory analysis, the samples were stored in insulated bags, and the temperature was controlled at below 5 °C. All samples were taken as parallel samples. Yantai CDC took 10% samples for sampling inspection, and used formula (1) to calculate the quality control coincidence rate.

1 $$ \mathrm{CR}=\left(\mathrm{A}1-\mathrm{A}2\right)/\left(\mathrm{A}1+\mathrm{A}2\right)\ast 2 $$

Among them, CR is the compliance rate, A1 is the nitrate nitrogen test result of parallel sample 1, and A2 is the nitrate nitrogen test result of parallel sample 2. If the absolute value of CR exceeds 5%, the data is considered an outlier, and the data of this group will be eliminated and will not be analyzed.

In this study, the proportion of runaway (|CR| > 5%) was 1.32%. The ratio of |CR| < 2% is 57.94%, and the ratio of 2% ≤ |CR| ≤ 5% is 40.74%.

### Study area

The study was conducted in rural areas of Yantai, Shandong Province of China. Yantai lies between Latitude − 37°32′ N and Longitude 121°24′E, covering a total area of 13,745.95 km^2^.Residents of rural areas in Yantai use shallow groundwater as drinking water, which is more susceptible to pollution by feces and fertilizers.

The rainfall in Yantai is concentrated in July–August, and its rainfall reaches 60% of the whole year. In addition, the amount of snowfall in Yantai from December to February is large, and its precipitation can account for about 25% of the year.

### Limits value of nitrate in drinking water

The World Health Organization’s limit for nitrate nitrogen is 11 mg/L.The study used the World Health Organization’s 2011 Drinking Water Hygiene Standard (Nitrate Nitrogen: 11 mg/L) for health risk assessment.

### Drinking water pathway

CDI can be expressed as the amount of potential toxins absorbed into the body through drinking water, and its formula is:

2 $$ \mathrm{CDI}=\mathrm{CW}\ast \mathrm{IR}/\mathrm{BW} $$

Among them, CDI indicates the chronic daily intake per unit weight [13], Cw is the average pollution concentration in water [14], IR is the water intake of human body, BW is the average body weight. The estimated values [15] are shown in Table 1 below.

**Table 1:** Calculation parameters used in the HHRA model

Variables	Implication	Value			Unit
		Adult male	Adult female	Juveniles	
IR	Water intake rate	2.0	2.0	0.67	L/d
BW	Average body weight	69.55	60.4	8.65	kg
SA	Surface area of contactable skin	1700	1600	3416.0	cm^2^
EV	Frequency of bathing	1	1	–	times/d
CF	Conversion factor	0.002	0.002	–	L/cm^3^
K_i_	Dermal permeability coefficient	0.001	0.001	–	cm/h

This data comes from the Ministry of Environmental Protection of China (2013)

### Dermal contact pathway

DAD can be used to estimate the amount of potential toxins absorbed by the human body through the dermal contact pathway, and its value represents the amount of chemical absorbed per unit of body weight, per day through the dermal contact pathway [16, 17]. Its calculation formula is:

3 $$ \mathrm{DAD}=\mathrm{CW}\ast \mathrm{Ki}\ast \mathrm{SA}\ast \mathrm{EV}\ast \mathrm{CF}/\mathrm{BW} $$

Among them, DAD is the daily dose absorbed via the skin (mg / kg·d), CW is the concentration of water pollutants (mg/L), Ki is the dermal permeability coefficient in water (cm/h), SA is the surface area of contactable skin (cm2), EV is Frequency of bathing (times/d), CF is the conversion factor (L/cm3), and BW is the average body weight (kg). See Table 1 for details.

### Description of risk characteristics

This study was based on the average daily intake nitrate content in the study area, and the risk characteristics were described by the hazard quotient (HQ). The hazard quotient is the most common method of calculating quantitative risk [18]. It is usually calculated as the ratio of exposure level to guideline value. The higher the hazard quotient, the higher the health risks involved, and a hazard factor of less than 1 is usually considered a low risk.

### Hazard quotient of drinking water pathways

The hazard quotient of health risk assessment for drinking water pathways is expressed as:

4 $$ \mathrm{HQoral}=\mathrm{CDI}/\mathrm{RFDoral} $$

Among them, HQoral is the hazard quotient ingested by oral drinking water, CDI is the chronic daily intake (mg / kg.d), and RfDoral refers to the reference dose (mg / kg.d).

In this study, the reference dose of oral nitrate nitrogen was 0.36 mg/kg.d (USEPA, 2001) and the reference dose of skin nitrate nitrogen intake was 0.18 mg/kg.d [19].

### Hazard quotient of dermal contact pathway

The hazard quotient of dermal contact pathway for nitrate health risk assessment is expressed as:

5 $$ \mathrm{HQderm}=\mathrm{DAD}/\mathrm{RFDderm} $$

Among them, HQderm is the hazard quotient of absorption through the skin, DAD is the absorbed dose of skin (mg / kg · d), RfDderm is the reference dose of skin absorption (mg / kg · d).

The total hazard quotient for nitrate health risk assessment is expressed as:

6 $$ \mathrm{HQ}=\mathrm{HQoral}+\mathrm{HQderm} $$

### Calculation formula for non-carcinogenic risk

After the nitrate in drinking water enters the human body, its health risks of non-carcinogenic risks are calculated by the formula.

7 $$ \mathrm{Rn}=\sum \mathrm{Rn}\mathrm{i}=\left(\mathrm{CDI}/\mathrm{RFDoral}\right)\ast 10-6/70+\left(\mathrm{DAD}/\mathrm{RFDderm}\right)\ast 10-6/70 $$

Among them, 70 is the average life expectancy of humans (a).

### Statistical analysis

The data was input into Excel 2003 for sorting, and the results were analyzed by SPSS19.0 statistical software. The statistics use descriptive analysis, *χ*^2^ test. *P* < 0.05 was considered statistically significant. Principal component analysis (PCA) was used to analyze the relationship between other chemicals in groundwater and nitrate concentration.

## Results

The sample sizes monitored in 2015, 2016, 2017 and 2018 were 836, 400, 572 and 540, respectively. The total number of monitoring in the 4 years is 2348. Among them, the qualified rate of nitrate nitrogen was 48.59%. According to the comparison of *χ*^2^ test results between different years, the difference of qualified rate of nitrate nitrogen in different years between 2015 and 2018 was not statistically significant (*χ*^2^ = 3.516, *P* = 0.319). See Table 2 for details.

**Table 2:** Test results of rural groundwater drinking water quality in Yantai from 2015 to 2018

Year	Nitrate nitrogen content (mg/L)	Number of samples	Qualified rate(%)
2015	23.4 ± 18.1	836	48.33
2016	23.2 ± 18.2	400	49.50
2017	27.1 ± 21.1	572	45.80
2018	15.7 ± 14.4	540	51.30
Total	21.9 ± 18.3	2348	48.59

One thousand one hundred seventy-four water samples were monitored during the dry and rainy seasons, respectively. The nitrate nitrogen qualified rate were 48.98 and 46.76%, respectively. There was no statistically significant difference in the qualified rate of nitrate nitrogen at two different periods(*χ*^2^ =1.154, *P* = 0.283). See Table 3 for details.

**Table 3:** Test results of different sampling periods in rural areas of Yantai from 2015 to 2018

Water type	Nitrate nitrogen content (mg/L)	Number of samples	Qualified rate(%)
Dry season	21.6 ± 18.1	1174	48.98
Wet season	22.2 ± 18.2	1174	46.76
total	21.9 ± 18.3	2348	48.59

### Correlation matrix of six factors

Using SPSS for principal component analysis, the following Table 4 can be obtained:

**Table 4:** Correlation matrix (linear relationship) between various chemical parameters

Variables	Nitrate	Dissolved solids	Total hardness	Chloride	Sulfate	Ammonia nitrogen
Nitrate	1					
Dissolved solids	0.6870	1				
Total hardness	0.6852	0.9210	1			
Chloride	0.5277	0.8382	0.7854	1		
Sulfate	0.3252	0.5753	0.5550	0.4490	1	
Ammonia nitrogen	0.0237	0.1355	0.1083	0.1031	0.0582	1

### Nitrate content distribution in well water

The nitrate concentration in the well water of this study ranged from 0.075 to 166.4 mg / L, the arithmetic mean was 21.9 mg / L, the median (C_EXP50_) was 17.8 mg / L, and the high exposure (C_EXP95_) was 53.4 mg / L.

### Exposure assessment of nitrate in adult population

Based on the C_EXP_ value and adult drinking water intake, formula (2, 3) was used to calculate adult nitrate intake. The hazard quotient (HQ) is further calculated using formula (4,5,6). The results are shown in Table 5. It can be seen from Table 5 that most adults (both male and female) in Yantai rural areas have taken in an excess of nitrate from the perspective of drinking water, and their hazard factor (HQ) exceeds 1. This study used the US Environmental Protection Agency’s standard of 1.6 mg / kg • d, which is widely used in developed countries. Because its nitrate content is measured by nitrate, it is necessary to convert into China standard nitrate (in terms of nitrogen), and its daily allowable intake (CDI) is 0.36 mg/kg/d.

**Table 5:** Summary of risk data for exposure to different drinking water nitrate levels

Different groups	HQ_oral50_	HQ_oral95_	HQ_derm50_	HQ_derm95_	HQ_50_	HQ_95_	Rn_50_	Rn_95_
Adult male	1.43	4.27	0.0048	0.0146	1.43	4.28	2.05*10^− 8^	6.12*10^− 8^
Adult female	1.65	4.91	0.0053	0.0155	1.66	4.93	2.36*10^−8^	7.04*10^− 8^
Juveniles	3.85	11.49	–	–	3.85	11.49	5.50*10^−8^	1.64*10^−7^

### Exposure assessment of nitrate in juveniles

It can be seen that the high-risk group health risk (HQ_95_) of juveniles is at a higher risk, with an HQ_95_ of 11.49 and an HQ_50_ of 3.85, both significantly higher than 1. Juveniles have a higher health risk than adults. As shown in Table 5 .

## Discussion

From 2015 to 2018 in Yantai, the qualified rate of nitrate in rural groundwater was only 48.59%. According to the monitoring data of drinking water quality in rural areas of Shandong Province, the level of nitrate in the Jiaodong area, including Yantai, Weihai and other cities, is obviously higher than other cities, which may be related to the geological environment, and it may also be related to the large amount of fertilizer used to grow fruit trees. In this study, there was no significant change of nitrate content during dry season and wet season, and there was also no significant change between 2015 and 2018, which indicated that nitrate content didn’t change with time and its content is relatively stable.

There is little information on the adverse effects of nitrate on adult health. Most researches focus on health risk analysis of toxicological indexes such as fluoride in drinking water. Some studies have shown that there is a link between the large intake of nitrate and thyroid dysfunction, gastrointestinal cancer [2]. A study [3] showed that women who used high nitrate drinking water in two states in the United States, had offspring with birth defects. The internationally recommended acceptable risk level is between 1 × 10-6a^− 1^ and 1 × 10-4a^− 1^, and the risk level higher than 1 × 10-4a^− 1^ is considered unacceptable. The risk level lower than 1 × 10-6a^− 1^ is considered acceptable, while the risk level of 1 × 10-5a^− 1^ is moderate. In this study, only one toxicological index of nitrate was analyzed, and the Rn value of juveniles reached a high level (1.64*10^− 7^).

In this study, the hazard quotient (HQ) of both adults and juveniles was more than 1. Adult females are more vulnerable to groundwater nitrate pollution than adult males, but the difference is not significant. In this study, the exposure risk of juveniles is the highest, even if the dermal contact pathway is not considered, the HQ value of juveniles is significantly higher than that of adults. This indicates juveniles are more likely to be affected by health risks from exposure to nitrates in drinking water. Due to the special physiological characteristics and behavioral habits of juveniles, and as their bodies are in a developmental stage, their gastrointestinal absorption of certain substances increases, which makes them more vulnerable to external pollutants [20].

This study considers the two nitrate exposure pathways through drinking water and dermal contact. It can be seen that there is a large gap between the two exposure pathways. The HQ value caused by drinking water is much higher than that of the dermal contact pathway. It can be seen that drinking water is still the main route of nitrate exposure in this area, including direct drinking water and water in food intake.

This study used principal component analysis to provide a correlation matrix between variables and considered all the variables mentioned (multiple relationships). A significant correlation coefficient (R2) is defined as 0.5 [21]. It can be seen that nitrate is positively correlated with total dissolved solids, total hardness, and chloride, but not with ammonia nitrogen. The correlation between nitrate and total dissolved solids may be related to geological factors, and it may also be related to the use of fertilizers. The positive correlation between nitrate and the total hardness may be related to the large-scale application of fertilizers in crop production [22], and it could also be related to geological factors. Positive correlation between nitrate and chloride may be related to the pollution effects of feces and livestock waste [23]. In agricultural production, the use of chemical fertilizers or the pollution by feces increases the concentration of calcium and chlorine ions in groundwater, which may be part of the reason for nitrate exceeding the standard. But this does not explain the fact that nitrate content in the western part of Shandong is lower than that in the eastern part of Shandong, and chemical fertilizer is also widely used in the western part of Shandong. By studying the correlation between the main chemical components of groundwater and nitrate content, this study reveals that high concentrations of nitrate in this area may be caused by the interaction of groundwater and soil rocks and the application of fertilizers in agriculture.

### Limitations of the study

In this study, there was a lack of calculation parameters for the exposure of bath water for juveniles, so this study did not consider the entry of nitrate through the dermal exposure pathway [24].

This study adopted the US Environmental Protection Agency’s 1.6 mg/kg.d as the daily nitrate intake standard, This standard has been adopted in developed countries, but its applicability to China is uncertain.

## Conclusions

In the study, the nitrate concentration in the wells in rural areas of Yantai ranges from 0.075 to 166.4 mg/L. 63.3% of the monitoring points exceeded the WHO guidance value of 11 mg/L. The nitrate content in rural areas of Yantai is relatively stable and will not easily change over time. The intake of nitrate in well water of adults has a certain risk, while the intake of nitrate in well water of juveniles has a higher risk. The higher level of groundwater nitrate in the rural areas of Yantai may be caused by the combination of geological factors and chemical fertilizers.

## Data Availability

The datasets used and/or analyzed during the current study are available from the corresponding author on reasonable request.

## Abbreviations

CDC: Center for Disease Control and Prevention
GB/T: Standard Test Method for Drinking Water of China
CR: Compliance rate
CDI: Chronic daily intake per unit weight
Cw: Concentration of water
IR: Water intake rate
BW: Average body weight
DAD: Daily dose absorbed via the skin
Ki: Dermal permeability coefficient
SA: Surface area of contactable skin
EV: Frequency of bathing
CF: Conversion factor
HQoral: Hazard quotient ingested by oral drinking water
HQderm: Hazard quotient of absorption through the skin
Rn: Health risks of non-carcinogenic
C_EXP50_: Median exposure concentration
